# Viral Eco-Genomic Tools: Development and Implementation for Aquatic Biomonitoring

**DOI:** 10.3390/ijerph19137707

**Published:** 2022-06-23

**Authors:** Gomaa Mostafa-Hedeab, Abdou Kamal Allayeh, Hany Abdelfattah Elhady, Abozer Y. Eledrdery, Mobarak Abu Mraheil, Ahmed Mostafa

**Affiliations:** 1 Pharmacology Department and Health Research Unit, Medical College, Jouf University, Skaka 11564, Saudi Arabia; 2 Water Pollution Department, Virology Laboratory, National Research Centre, Dokki, Giza 12622, Egypt; drallayeh@yahoo.com; 3 Surgery Department, Medical College, Jouf University, Sakaka 11564, Saudi Arabia; haelhady@ju.edu.sa; 4 Department of Clinical Laboratory Sciences, College of Applied Medical Sciences, Jouf University, Sakaka 11564, Saudi Arabia; ayelderdery@ju.edu.sa; 5 German Center for Infection Research (DZIF), Institute of Medical Microbiology, Justus-Liebig University, 35392 Giessen, Germany; 6 Center of Scientific Excellence for Influenza Viruses, National Research Centre, Giza 12622, Egypt

**Keywords:** enteric viruses, aquatic biomonitoring, eco-genomic tools, pollutants

## Abstract

Enteric viruses (EVs) occurrence within aquatic environments varies and leads to significant risk on public health of humans, animals, and diversity of aquatic taxa. Early and efficacious recognition of cultivable and fastidious EVs in aquatic systems are important to ensure the sanitary level of aquatic water and implement required treatment strategies. Herein, we provided a comprehensive overview of the conventional and up-to-date eco-genomic tools for aquatic biomonitoring of EVs, aiming to develop better water pollution monitoring tools. In combination with bioinformatics techniques, genetic tools including cloning sequencing analysis, DNA microarray, next-generation sequencing (NGS), and metagenomic sequencing technologies are implemented to make informed decisions about the global burden of waterborne EVs-associated diseases. The data presented in this review are helpful to recommend that: (1) Each viral pollution detection method has its own merits and demerits; therefore, it would be advantageous for viral pollution evaluation to be integrated as a complementary platform. (2) The total viral genome pool extracted from aquatic environmental samples is a real reflection of pollution status of the aquatic eco-systems; therefore, it is recommended to conduct regular sampling through the year to establish an updated monitoring system for EVs, and quantify viral peak concentrations, viral typing, and genotyping. (3) Despite that conventional detection methods are cheaper, it is highly recommended to implement molecular-based technologies to complement aquatic ecosystems biomonitoring due to numerous advantages including high-throughput capability. (4) Continuous implementation of the eco-genetic detection tools for monitoring the EVs in aquatic ecosystems is recommended.

## 1. Introduction

The aquatic ecosystem, including coastal waters, rivers, and lakes, is continuously menaced by the infiltrations and drainage of anthropogenic wastewaters. Consequently, this environmental hazard requires the perpetual and constant implementation of up-to-date monitoring systems to become more comprehensive, perspicuous, and specific to targeted pollutants [1]. Recently, genomic-based tools substituted the conventional burdensome morphological monitoring tools and vastly implemented as a routine tool in cutting-edge research and biotechnologies. However, the application of the modern genomics tools in environmental biomonitoring is so far considered an innovative approach and demands improved knowledge to be applied in aquatic biomonitoring. Until now, environmental genomics was mainly used to screen known morphologically distinguishable bio-indicator taxa [2].

**Figure 1:**
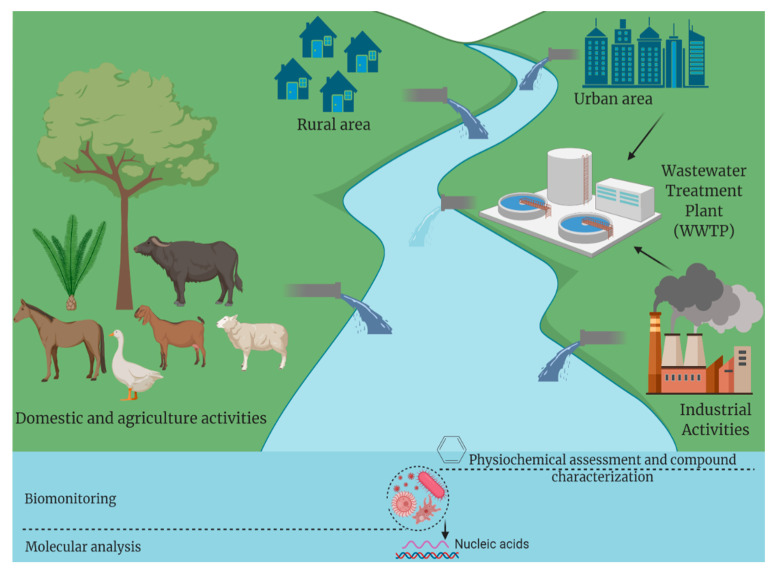
Sources of anthropogenic pollutants and current approaches for aquatic biomonitoring. The figure is assembled using dynamic BioRender assets.

Enteric viruses (EVs) occur naturally in their infective (active) form in aquatic environments and are commonly introduced together with bacterial and parasitic microbes through anthropogenic activities, including agricultural runoff, urban runoff, leaking sewage and septic systems, sewage outfall and vessel wastewater discharge (Figure 1). Being transmitted through the fecal–oral route, EVs replicate usually and primarily in the epithelial cells of the host gastrointestinal (GIT) tract and are secreted in the feces of infected patients in extremely high numbers (10^5^ and 10^11^ viral particle/gram of stool) [3]. Besides anthropogenic activities, EVs are commonly secreted indirectly into in aquatic groundwater, rivers, aerosols discharged from sewage/wastewater treatment plants, estuarine water, inefficiently treated water, and drinking water receiving untreated contaminated wastewater, and wastewater-contaminated private wells [3].

The clinical complications of EVs-infections are primarily associated to diarrhea and self-limiting gastroenteritis in infected humans, EVs can also trigger more life-threating complicated syndromes including respiratory tract (RT) infections, conjunctivitis, hepatitis, and diseases that have high severity and high fatality rates (e.g., aseptic meningitis, encephalitis, and paralysis) [4]. In addition, some EVs infections are associated with chronic disorders, e.g., insulin-dependent diabetes mellitus (type 1 diabetes) and inflammatory cardiomyopathy (known as myocarditis) [5]. On the other hand, infections with enteric viruses are commonly asymptomatic in domestic animals (e.g., cattle and swine), but can sometimes lead to unpleasant economic losses such as abortion and diseases of the central/peripheral nervous system of the animal (neurological disorders) [3,6].

This review article provides an insight to available and implemented detection tools to monitor multispecies viral pathogens, including EVs and non-EVs to provide full water-based epidemiology and biomonitoring. The accurate and precise detection of viral pathogens in surface or wastewater samples can positively provide necessary information for controlling the source of pollution, defining human-related health risks, and possible zoonotic and reverse zoonotic events [3]. This also impacts public heath by understanding the prevalence of human and non-human EVs and facilitating the documentation of viral pathogens for water quality assessment tools and library-independent source tracking [7].

## 2. Enteric Viruses in Water and Their Impact on Public Health 

There are more than 200 recognized EVs classified in at least 13 viral families, among which 140 serotypes are known to infect humans causing diseases of diverse symptoms and variable severity [8]. EVs are usually transmitted using the fecal–oral route and primarily infect the GIT of the host efficiently, whether human or domestic animal, leading to virus shedding in relatively high amounts in their feces [9]. Upon transmission, EVs are commonly associated with gastroenteritis (mild and localized infection) or serious acute illnesses, including infections of the central nervous system (meningitis, encephalitis, and poliomyelitis), respiratory diseases, conjunctivitis, and non-specific febrile illnesses [3]. Moreover, EVs have also been connected to the aetiology of some chronic diseases, e.g., chronic fatigue syndrome and diabetes mellitus [10,11]. EVs, mainly result in waterborne infections to humans and animals, including noroviruses (NoVs), hepatitis A and E viruses (HAV and HEV), rotaviruses (RVs), adenoviruses (Ads), and enteroviruses [11].

Common types of EVs of particular interest regarding their epidemiology and pathogenicity in humans belong to the following families: (a) Picornaviridae (coxsackieviruses, polioviruses, enteroviruses, and echoviruses); (b) Adenoviridae (adenoviruses); (c) Caliciviridae (NoVs, astroviruses, and caliciviruses); and (d) Reoviridae (reoviruses and RVs). The characteristics of EVs, along with the associated health risk are summarized in Table 1.

Unlike enveloped viruses, most EVs are non-enveloped viruses of distinct cellular and molecular structures making them more resistant to many natural disinfection factors including slow sand filtration, infiltration/percolation in the soil, drying out and/or heat, and less tolerant to conventional viral removal water treatment technologies including ultraviolet (UV) irradiation and active chlorine, chlorine dioxide, ozone, and peracetic acid. To this point, advanced technologies have been applied in wastewater and drinking water treatment plants including combinations of ozone and hydrogen peroxide, or ozone and UV radiation, or hydrogen peroxide and UV radiation, or UV radiation with titanium dioxide and finally advanced membranes technologies [12]. However, none of these disinfection processes can guarantee complete removal of EVs in treated water if the water comes from unsuitable sources with high loads [13,14]. To this point, EVs are considered to be emerging waterborne pathogens and difficult to remove completely in wastewater treatment plants (WWTPs). For instance, (1) circoviruses are non-enveloped viruses with single-stranded circular DNA and are resistant to heat inactivation; (2) picobirnaviridae are small non-enveloped viruses with double-stranded RNA and are extremely resistant to UV light inactivation; (3) parvoviruses are the smallest EVs with a single-stranded RNA (ssRNA) genome and high heat resistance; (4) polyomaviruses are non-enveloped double-stranded DNA (dsDNA) viruses and are very heat stable but less resistant (relatively sensitive) to chlorination than enteroviruses [3]; and (5) adenoviruses are double-stranded DNA (dsDNA) and are very resistant to UV.

EVs have been found in drinking water, surface water, groundwater, seawater and treated and untreated wastewater in several countries. Surveillance programs to monitor these EVs in the predefined aquatic systems is therefore critical for accurate risk assessment and further management. Nevertheless, individual detection of known viral aquatic contaminants (>100) is impractical. Therefore, viral indicators are predominantly applied for the long-term monitoring of viral contamination in freshwater, wastewater, and marine environments, and quantitatively estimate wastewater viral contaminants. Typical viral indicators must be easily detectable and quantifiable, source-specific, resistant to wastewater treatment processes, and persistent, intact, and infectious in the aquatic systems with high prevalence [15]. Several viruses were suggested as viral indicators including Aichi virus and human mastadenoviruses to indicate human-derived contamination and contamination by domestic wastewater due to their easy detection, culturability in wastewater and in the polluted environment [15]. Similarly, human noroviruses (HuNoV) are found to be present in extremely high concentrations in infected human feces (Up to 10^11^ log_10_ genomic copy numbers (GC)/g) [16]. Being associated with secreted feces, HuNoV may accordingly be accumulated in high concentrations in wastewater [16]. Following wastewater treatment with mechanical systems and chlorine disinfection, a significant reduction in NoV concentrations occurred for the main two genotypes (GI and GII) to be in the range between 2.53 and 5.9 log_10_ GC/liter [16], suggesting that noroviruses are applicable as indicators of anthropogenic pollution [17].

Biomonitoring (BM) is commonly known as the act of observing and assessing the state and ongoing changes in ecosystems and components of biodiversity and landscape, including the types of natural habitats, populations, and species [18]. Therefore, BM in many settings has been limited in scope to living aquatic animals and chemicals pollutants rather than new freshwater emerging pollutants (e.g., microplastics) and important taxonomic groups (e.g., microbes and viruses). During the last two decades, the idea that genetic analyses of viral pathogens can advantageously replace conventional cell culture-based and electron-microscopic-based morphological methods for typing and subtyping has significantly increased. To this point, metabarcoding was developed as a set of techniques to identify total viral loads/types/subtypes simultaneously from an environmental sample with standardized viral genomic-based tools [19]. This resulted in broadening of the environmental and aquatic biomonitoring and led to the emergence of the concept of “Biomonitoring 2.0” (BM-2.0). This approach introduces novel perspectives for monitoring environmental communities, including viral pathogens [20]. To apply a specific detection method for viral pathogens biomonitoring, the method must be appropriate, optimized against possible limitations, verified for its applicability, reproducibility, sensitivity, specificity and, if possible, cost- and time-effective [21].

**Table 1:** Transmissible viruses via water.

Family	Genus	Virus Name	Genome	Size (Nm)	Types	Disease	Ref.
**Picorna-viridae**	Hepatovirus	Hepatitis virus	+ssRNA	27–32	3 types	Hepatitis	[22]
	Kobuvirus	Aichivirus	+ssRNA	30	6 types	Gastroenteritis	[23]
	Enterovirus	Poliovirus	dsDNA	40	14 species	Paralysis, aseptic meningitis	[24]
		Coxsackievirus	+ssRNA	30	Cox A1-23 and B1-6	Myocarditis, aseptic meningitis, Bornholm disease and epidemic pleurodynia	[25]
		Echovirus	+ssRNA	30	28 types	Fever, rash, respiratory and heart disease, aseptic meningitis	[26]
		Enterovirus	+ssRNA	30	12 species	Gastroenteritis	[27]
	Parechovirus	HPeV	+ssRNA	28	19 genotypes	Gastroenteritis, respiratory and CNS diseases, and sepsis	[28]
	Cosavirus	HCoSV	+ssRNA	30	5 species	Gastroenteritis non-polio AFP	[29]
	Aphthovirus	FMDV	+ssRNA	25	7 serotypes	Respiratory diseases	[23]
**Adeno-viridae**	Mastadenovirus	Adenovirus (AdV)	dsDNA	70	60 types	- Respiratory disease - Eye infections	[30]
**Parvo-viridae**	Erythrovirus	Parvovirus	ssDNA	22	3 genotypes	Gastroenteritis	[31]
	Bocavirus	Bocavirus	ssDNA	20	4 genotypes	Respiratory diseases	[31,32]
**Reoviridae**	Rotavirus	Rotaviruses	dsRNA	80	9 species	Gastroenteritis	[33]
**Hepeviridae**	Hepevirus	Hepatitis E virus	+ssRNA	27–34	4 genotypes	Infectious hepatitis	[34]
**Picobirna-viridae**	Picobirnavirus	Picobirnavirus	dsRNA	35	Human and Rabbit Picobirna-virus	Respiratory diseases and gastroenteritis	[35]
**Astroviridae**	Mamastrovirus	Astrovirus	+ssRNA	35	8 serotypes	Gastroenteritis	[36]
**Bunya-viridae**	Hantavirus	Hantavirus	−ssRNA	120	4 genera	Hemorrhagic fever and cardiopulmonary syndrome	[37]
**Flavi-viridae**	Flavivirus	TBEV	+ssRNA	50	5 subtypes	Fever, meningitis and encephalitis	[38]
**Arena-viridae**	Arenavirus	Arenavirus	−ssRNA	40–200	4 genera	Aseptic meningitis and hemorrhagic fever	[39]
**Corona-viridae**	Alphacorona-virus	HCoV-229E	+ssRNA	120–140	7 subtypes	- Ranging from mild common cold to severe respiratory syndrome - Gastroenteritis	[11,40]
		HCoV-NL63					
	Betacoronavirus	HCoV-OC43					
		HCoV-HKU1					
		MERS-CoV					
		SARS-CoV					
		SARS-CoV-2					
**Orthomyxo-viridae**	Influenza A virus	AIVs (HSN1 and H9N2)	−ssRNA	100	Many subtypes	- Respiratory diseases - GIT symptoms	[41]
**Paramyxo-viridae**	Henipavirus	Nipah virus	−ssRNA	40	2 genotypes (M and B)	- Encephalitis - Inflammation of the brain	[42]
**Calici-viridae**	Norovirus	Norovirus	+ssRNA	27–40	9 genotypes	Gastroenteritis	[43,44]
	Sapovirus	Sapovirus	+ssRNA	27–40	18 genotypes	Gastroenteritis	

Abbreviations: Avian influenza viruses (AIVs); non-polio acute flaccid paralysis (non-polio AFP); human coronavirus (HCoV): tick-borne encephalitis virus (TBEV); human parechovirus (HPeV); human cosavirus (HCoSV); foot-and-mouth disease virus (FMDV); coxsackievirus (Cox); ribonucleic acid (RNA); positive sense single stranded (+ss); negative sense single stranded (−ss); double stranded (ds); reference (Ref).

## 3. Waterborne Viruses Concentration Methods

Enteric viruses are frequently found in quantities too tiny to detect directly. The identification of waterborne viruses is a multi-step process that begins with a viral concentration and concludes with a detection test [45]. The sole conceivable exception is sewage, where viruses may be present in sufficient load titers to be detected without concentration. Although enteric viruses may be identified in small amounts of sewage (up to 1 L), larger volumes (10–100 L) are often required for viral identification in other types of water. According to Block and Schwartzbrod [46], the ideal concentration method must meet a number of criteria in order to be practical, including being technically simple to implement in a short period of time, having a high recovery rate, concentrating a diverse range of viral types, and being economical, repeatable, and reproducible. No single approach, according to these criteria, can fulfil all these requirements. As a result, the viral concentration step is likely to consist of at least two phases. The first phase reduces the volume to between 100 and 400 mL, and the second phase from 2 to 10 mL. A variety of incredibly efficient methods for concentrating waterborne viruses have been established, and these are reviewed in detail here.

### 3.1. Adsorption/Elution Method

This method is frequently utilized and is based on electrostatic interactions in aquatic settings between electronegative viruses and either negatively or positively charged filters. The pH and ionic strength of the solution have an effect on viral adsorption using filters. As a result, when utilizing solutions capable of disrupting these linkages, such as glycine, beef extract, and polyethylene glycol (PEG), viral elution was most successful. This approach is less expensive and has a good recovery rate (60–74%) [46,47]. However, membranes are prone to clogging and cannot be used with even somewhat cloudy water or at high flow rates [48]. To overcome obstruction without having to change membranes or cartridges as frequently, the surface area of filtration must be increased by utilizing larger cartridges or filters.

### 3.2. Ultrafiltration

Ultrafiltration isolates viruses from other particles in water samples based on molecular weight differences by passing the liquid through capillaries, membranes, or hollow fibers. Adsorption of viruses to ultrafilters has been frequently associated to lower predicted viral recoveries. Several solutions have been successfully employed in ultrafilters as retentive volume additives to increase elution and/or back flushing efficiency. Because ultrafiltration does not require pre-conditioning, it is possible to recover a wide spectrum of viruses, including those that are sensitive to the pH changes. Recovery efficiency is typically good, although similar to other procedures in that it varies [49,50].

### 3.3. Ultracentrifugation 

Ultracentrifugation has been used for decades to concentrate waterborne viruses. This methodology recovers viruses at a rate comparable to or even greater than previous concentration approaches. For example, Prata and his colleagues showed an average viral recovery efficiency of 69% and 76% for wastewater and recreational water samples, compared with 38 and 22%, respectively, when using an organic flocculation technique [51]. Ultracentrifugation has several advantages over other methods of viral concentration, including lower material costs per sample because containers may be reused. Furthermore, no pH adjustments are required, and no elution or extra concentration operations are required; hence, ultracentrifugation takes less time than organic flocculation techniques and does not introduce any PCR inhibitory chemicals [51]. There are many substantial difficulties to using ultracentrifugation to concentrate viruses, including the initial expense of an ultracentrifuge unit and the fact that only tiny quantities (10 mL–1 L) can be reasonably handled [52,53].

### 3.4. Hydro-Extraction Method

Water samples are placed in a dialysis bag and exposed for many hours at 4 °C to polyethylene glycol (PEG). Water is drawn through the semipermeable barrier by the solid, but viruses and other macro solutes remain in the bag. The limitations include the sample amounts (up to 1 L) and the co-concentrated matrix component, which may include inhibitors for further analysis [54].

### 3.5. Freeze-Drying Technique

This approach uses a freeze and dry process to remove water from samples. This method recovered more rotavirus (45%) than the PEG precipitation method (17%). This approach is best used in combination with a primary concentration technique due to the small sample volume (mL) and relatively long waiting period (4 h) [55].

### 3.6. Antibody-Capture Technique

Immuno-magnetic separation concentrates waterborne viruses. The high specificity of antibody-based capture is advantageous. Due to the high cost of antibodies, the sample amount is limited to a few milliliters. As a consequence, rather than raw water, this approach was applied to purified concentrations after successive concentration operations [56].

## 4. Conventional Viral Detection Tools for Aquatic Biomonitoring

EVs detection in aquatic water is a complex trait because of the high dilution factor of the obtained samples making these viruses sporadically occurring in environmental samples. In addition, environmental samples contain a variety of naturally occurring inhibitory substances that may mask the presence of active viral particles in the samples. Electron microscopy and animal cell culture systems represented, for a long time, the standard method for detecting infectious viruses in water.

### 4.1. Electron Microscopy

Electron microscopy (EM) was heavily applied during the 19th century to recognize newly emerging viruses. Using EM, the first successful trial to discover the structure of poliovirus was achieved in 1952 [57], followed by norovirus (Caliciviridae) in 1972 [58]. The study of virus-host interactions started with the help of EM in the mid-1950s and subsequent virus classification was mainly dependent on the morphological features, revealed through EM examination [59].

Despite that EM is considered by many researchers to be an old technique, especially with the availability of highly specific and sensitive molecular diagnostics, EM is a fundamental tool to detect the etiological agent of new or unusual outbreaks caused by emerging and potential bioterrorism viruses. Moreover, EM is currently on the forefront in the field of structural virology especially in studying the correlation between viral ultrastructure and the clinical viral diagnoses and pathogenesis. Moreover, in the research area, EM is highly applied in different modalities including electron tomography, immunoelectron microscopy, and cryo-electron microscopy to study the viral fitness, cellular cascades which are involved in virus replication, and viral-replication cycle [59].

EM/bioinformatics combination offers the transition from 2D imaging to 3D remodeling which enables structural and functional analyses that broaden our knowledge of the spectacular diversity in viral-particle structure and replication cycle. Together with confocal laser scanning (CLS) microscopy, EM allows live imaging of infected and control cells with high-resolution analysis [60]. One main advantage of using EM over other molecular and serological methods for viral diagnosis is that EM does not require virus-specific materials for recognition. However, EM can only identify a virus through morphology, making it impossible to identify a virus beyond the family level [59].

### 4.2. Cell Culture Systems

Since the early 1960s, cell culture systems were established and routinely used for virus isolation and viral disease diagnosis. Therefore, the cell culture approach was described for decades as the “gold standard” for viral diagnosis. However, the cell culture approach for diagnosis is relatively time-consuming and requires considerable technical expertise. Additionally, cell cultures allow only active viruses to grow. Simultaneously, viruses require specific permissive and susceptible cell lines (Table 2) which may be difficult to manage in the case of environmental samples, where a collection of viral and bacterial pathogens is expected to exist together.

With the innovation of non-cell culture methods for the rapid identification of viral nucleic acids and antigens, the importance of viral culture for viral diagnosis retreated [79]. In combination with electron microscopy and immunofluorescence (IF) techniques, the sensitivity of cell culture models has been increasingly improved. Nevertheless, these staining techniques cannot be implemented to definitively characterize all viruses and distinguish their different subtypes/genotypes.

### 4.3. Immunological Methods

Most immunological methods for EVs rely on antigen detection such as enzyme immunoassay (EIA), latex agglutination test, or immunochromatography technologies. For instance, the latex agglutination reaction (LAT) was developed in the 1980s to detect bacterial toxin A. LAT assays identify the observed clumping ability when a filed sample comprising a specific antigen is mixed with latex particles with a specific antibody coat on their surface, leading to agglutination. The LAT assay is considered one of the more favored, rapid, and easiest methods. Therefore, it is used currently to rapidly provide a fast diagnosis tool for the identification of several EVs under laboratory and non-laboratory settings. Specifically, in different virology laboratories in developing countries, the LAT assay is so far reputedly used to diagnose EVs in specimens such as human EVs (e.g., rotaviruses, noroviruses, astroviruses, and adenoviruses) and animal EVs [58,80]. Being qualitative with limited sensitivity and specificity, and unable distinguish between infective and defective viral particles, the results of these techniques are questioned.

The enzyme-linked immunosorbent assay (ELISA) is known to be one of the most commonly used and easy-to-go serological diagnostic assay. Interestingly, numerous modifications and updates of this technique are currently applied and available as commercial kits to detect EVs. For instance, a fully automated and ultrasensitive bioluminescent enzyme immunoassay (BLEIA) was introduced to detect norovirus capsid antigen. This technique is as sensitive as ELISA in the detection of various EVs (reviewed in [58]). More recently, a workable sensitive sandwich ELISA to detect norovirus genogroup II (NoV-II) was developed as an applicable assay for NoV-II early stage diagnosis with improved sensitivity [81]. 

### 4.4. Biosensors

Biosensors are ready-to-use measurement devices that can sense several environmental biomolecules. These devices are currently applied on large scale for the detection of clinical pathogens including viruses [82]. More recently, nanotechnology revolutionized the biosensors in terms of device design and performance via developing nanoparticles that improve these sensors affinity, selectivity, and efficacy in detecting these viral pathogens [83]. Interestingly, biosensors are portable bioanalytical devices which mainly consist of an analyte, receptor, transducer, and signal reader to detect any biochemical interaction [82]. Biosensors have been used for detection of viruses in water, clinical samples, and food [82].

Fortunaly, biosensors are improved to enable virus detection in few minuteshour with high sensitivity and specificity. Moreover, the fast detection analyses of environmental samples using biosensors are cheap compared with other molecular methods, making it an ecomomic detection tool. Compared with PCR-based techniques, biosensors are not affected by inhibitors of molecular methods, which are extensively present in the concentrated water samples [84]. Several approaches for the development of biosensors have been conducted to detect a variety of waterborne viruses including norovirus, rotavirus, coronavirus, and influenza subtypes H3N2, H1N1, and H5N1 viruses [82,85].

Recently, biosensors concepts have been implemented to inaugurate Lab-On-Chip (LOC) or Point-of-Care Testing (POCT) devices to rapidly detect viruses and for viral disease diagnosis such as microfluidic chips that integrate many laboratory functions on a single chip and combining micro-electro-mechanical systems together with microfluidic technology [86].

These microfluidic devices have been recently advanced, attracted attention, and made its breakthrough in shortening the time and speed for virus detection. This technology can also significantly adapt virus testing for Point-of-Care in home settings. However, microfluidic chips face challenges for virus detection including: (1) collection and sample preparation integration, (2) application of quantitative methods, (3) and capacity for throughput and multiplex during outbreaks.

## 5. Viral Genetic Tools for Aquatic Biomonitoring

### 5.1. Polymerase Chain Reaction (PCR), Sequencing and Phylogenetic Analysis

Despite that cell-culture detection methods are considered the gold standard to isolate and further detect the infectious viral particle from aquatic samples; viral genetic-based molecular techniques became an optimum alternative to rapidly detect the virus in the sample within few hours with less cost. Commonly, most virology laboratories develop and implement polymerase chain reaction (PCR) and/or reverse transcriptase-polymerase chain reaction (RT-PCR) as well-established methods to detect DNA- and RNA-waterborne viruses, respectively. Compared with cell culture, both forms of this conventional PCR reactions are useful in detecting EVs in water samples due to their high specificity and sensitivity, especially in the detection of those viruses with low concentration in water samples and also non-culturable viruses [87]. 

Unlike cell culture for waterborne viruses, the PCR method underestimates the true level of contamination due to the usage of highly specific primers to capture the viral genome [87]. For instance, EVs in 4 (8%) out of 50 household wells were detected by PCR method, while no viruses were detected after inoculation of cell cultures [88]. 

The main limitations of the PCR technique is its inability to quantify viruses and also that it cannot define whether the detected EVs in the sample is infective or defective. Therefore, to improve the sensitivity, specificity and efficiency of PCR, updates of the PCR technique including integrated cell culture PCR (ICC-PCR), multiplex PCR, nested PCR (and semi-nested), real-time PCR (RT-PCR) (for quantification), digital PCR (dPCR), and droplet digital PCR (ddPCR) were introduced. Nested and semi-nested PCR methods were developed and implemented to augment the sensitivity and specificity of the PCR techniques with an internal primer through two run reactions of PCR. Nested PCR assays for adenoviruses were shown to significantly increase compared with conventional PCR assay with sensitivity limits of 10^−2^ adenovirus particle/mL [89]. Unfortunately, the improved high sensitivity of the nested PCR may be accompanied with a high probability of subsequent contamination when PCR products from the first round of PCR are transferred to second round of nested PCR [90,91].

On the other hand, real-time PCR (rt-PCR) or quantitative PCR (qPCR) is currently the most significant and widely used for virus detection in water in all environmental virology labs using either a SYBR Green or Taqman probe. Unlike the predefined techniques, qPCR is characterized by rapidity, sensitivity, reduction in contamination risk, and ability to provide quantification of viruses in samples. To the last point, the qPCR assay demands a curve of known concentrations of the standard genome. Using rt-PCR in combination with genotype or subtype specific-primers, it can be used for genotyping of waterborne viruses in the same reaction. Therefore, several studies have used rt-PCR to investigate viral outbreak in water and wastewater [92]. Previous studies have detected various viral species and viral loads of adenoviruses and EVs by rt-PCR/qPCR compared with the predefined methods [93]. The critical limitations of qPCR are similar to conventional PCR, including the inability to determine the viral infectivity in the samples and the probability of inhibition by inhibitor substances in wastewater samples that may affect qPCR amplification and lead to false negative results.

Since the emergence of Severe Acute Respiratory Coronavirus 2 (SARS-CoV-2) in 2019, the adoption of dPCR in detecting and quantifying viral pathogens in clinical, environmental, and wastewater surveillance samples has been accelerated. Uniquely, dPCR technologies apply Poisson distribution to estimate the most probable number (MPN) of a genetic target based on the endpoint fluorescence in each individual partition, without the need for a calibration curve. Compared with qPCR, well-optimized dPCR assays are capable of low inhibition rates, better sensitivity, less variation at the quantitative limit, and increased accuracy [94].

Following PCR-based techniques, nucleotide sequencing and phylogenetic analysis are performed to deeply characterize the amplified viral genetic amplicons (PCR products). Molecular genetic analyses of viruses in water and wastewater samples commonly reveal that EVs found in environmental samples harbor a genetically diverse viral genome (quasispecies) [95]. By sequencing and phylogenetic analysis of the PCR products, the major genotypes of viruses in aquatic and environmental samples are determined [96].

On the same hand, the identification of various antigenic subtypes and/or multiple types of EVs in a single run is always preferred and economic to solve the complex etiology and diversity posed by different EVs. Therefore, a multiplex RT-qPCR assay was innovated and implemented to detect diverse EVs (e.g., astroviruses, adenoviruses, rotaviruses, sapoviruses, and enteroviruses) [58,97]. Unfortunately, a multiplex RT-qPCR assay detected norovirus [98].

### 5.2. Isothermal Nucleic Acid Amplification-Based Assays

Recently, isothermal amplification (IA) methods including nucleic acid sequence-based amplification (NASBA), loop-mediated isothermal amplification (LAMP), single primer isothermal amplification (SPIA), and recombinase polymerase amplification (RPA), are developed for detecting genetic materials of contaminating pathogens including viruses from environmental samples using simpler (economic), rapid, specific, and sensitive techniques (Table 3). However, these techniques are not yet established in most central virology laboratories, especially in developing countries.

### 5.3. DNA Microarray Technology

DNA microarrays are microscope slides on which thousands of immobilized individual DNA capture fragments are spotted to hybridize with complementary target sequences in the organism of interest. Interestingly, microarrays approaches can detect multiple viruses (≥10,000 pathogens) using pathogen-specific complementary probes to hybridize target sequencing. Probe-genome hybridization is commonly observed using a reporter fluorescence [106]. 

Additionally, the inclusion of different “probe” sequences is capable of providing a tool for simultaneous detection of different types and subtypes. To these points, microarrays are potentially considered as a powerful technology for virus detection with a high specificity degree. However, the sensitivity is relatively low and the test cost is high. Therefore, they are unlikely to be implemented shortly in routine biomonitoring of environmental water samples.

Experimentally, microarrays were applied in environmental studies and evaluated for detection of EVs in complex environments. Microarrays were shown to provide a great potential as a specific, sensitive, and quantitative detection tool for EVs in environmental samples [107]. Similarly, microarrays have been applied with PCR for the detection and identification of rotaviruses, norovirus, human coronaviruses in wastewater [108], norovirus, HAV, rotavirus, adenovirus, astrovirus, and coxsackieviruses A and B [109].

Moreover, microarrays were applied in viral genotyping to determine the genotyping of norovirus in environmental and tap water. In addition, microarrays contribute to detect viral pathogens in environmental surface water in pandemic situations (e.g., SARS-CoV-2), where microarrays allowed scientists to determine the persistence of active SARS-CoV-2 virus for days [110]. Although it is a very good technique, its current application is limited due to its high cost. This technique should now be provided attention in view of its reliability and performance and considering its modulation into a cost-effective assay.

### 5.4. Next Generation Sequencing (NGS) and Metagenomic Technology

In the past decade, metagenomics and NGS have revolutionized the virological sciences including virus discovery and are widely employed by researchers in diagnostics and research laboratories. Both provide a package service for detection and sequence identification of total viral types and subtypes via analysis of total nucleic acids which are occurring in a complex biological matrix.

Based on their ultra-high throughput, scalability, and speed, viral metagenomics and NGS offer an optimum alternative approach to the predefined molecular method. Recent advances in NGS technology and metagenomics approaches facilitated the discovery of new viruses and other emerging microorganisms in environmental samples. Unlike bacteria in which metagenomics describe the diversity of the 16S ribosomal RNA, viral metagenomics describe the full or partial genomes of all viruses present in the sample.

Since 2002, viral metagenomics technology were used to determine viral species in environmental samples including freshwater, marine sediment, soil, and the human gut [87]. Traditionally, the viral metagenomic studies relied on standard cloning protocols of viral samples and then sequencing by Sanger technology [111]. More recently, the innovation and implementation of novel NGS platforms such as pyrosequencing, Ion Torrent, Illuminia, MinION, and ABI/Solid enabled high-throughput sequencing of RNA/DNA amplicons from known and newly emerging viruses in water and facilitated the discovery of these emerging and reemerging pathogens [87]. In combination with bioinformatics, NGS can translate a large amount of genomic data into extra knowledge regarding viral genomes [112].

Viral metagenomics application for aquatic biomonitoring of environmental water and wastewater provide an excellent platform to detect unknown waterborne viruses and/or EVs. In a study of sewage samples, authors identified 21 viral families, including several human DNA/RNA viruses such as *Picornaviridae*, and *Papillomaviridae* [113]. Recently, several viral pathogens were discovered in water by metagenomic technologies in river water samples [114,115]. Despite that NGS is considered a powerful tool, NGS-based metagenomic studies were limited mainly with three major challenges: (1) sample preparation for high-throughput sequencing, (2) contamination, and (3) specialized bioinformatic analysis.

Owing to the high data output of these NGS platforms, data processing, and analysis of these projects generally require strong computational infrastructure and technological expertise [112]. For instance, data analyses include the clustering of millions of viral genomic reads into many different separate genomes [116], and identifying these assembled genomes can be hampered by miss-annotations and incomplete reference databases [117]. The sequence analysis and processing tasks are computationally intensive and generally require dedicated bioinformatics expertise. Conclusively, data output from NGS platforms need workflow to be able to be analyzed. Commonly, the majority of NGS workflow consists of up to five different steps including: (1) assess the data quality, (2) filter, (3) mapping reads, (4) and annotation to reference databases.

On the other hand, there are several challenges in analyzing viral metagenomes including: (1) high output of sequencing reads; (2) assembly of millions of genomic fragments; (3) annotation of all assembled genomes to reference databases; and (4) metagenomic data interpretation. To sum, this technique should now be provided a attention in view of its reliability and performance, and considering its modulation into a cost effective assay.

### 5.5. ChIP-Seq Analysis

Chromatin immunoprecipitation (ChIP) followed by NGS sequencing (ChIP-Seq) is not purely directed to viral diagnosis, it is used to define the interaction between the virus proteins including those for enteric viruses and the genomic DNA. ChIP is an essential technique to study viral protein-genome interactions within the infected cell. This technique includes major steps such as cell fixation, sonication, immunoprecipitation, and sequencing of the immunoprecipitated DNA. 

Using the ChIP-Seq approach, our knowledge about the genome loci interactions in host cell with EVs was clarified [118,119,120]. Using specific antibodies against viral DNA and/or RNA-associated antigens, ChIP-Seq analysis can be developed and implemented for diagnosis of viral pathogens. However, this technique is time consuming and needs special training on different approaches such as immunoprecipitation and sequencing of the immunoprecipitated genetic motifs.

To sum, each viral pollution detection method has its own merits and demerits (Table 4). Therefore, it would be advantageous for viral pollution evaluation to be integrated as a complementary platform.

## 6. Conclusions

Aquatic biomonitoring is an emerging approach that simply aims to the observation and assessment of the constant and ongoing alterations in aquatic ecosystems. Therefore, biomonitoring falls between community health surveillance and environmental monitoring. Constantly, scientific research is improving the detection of underlying resources for water pollution with enteric viruses (EVs) via conventional or revolutionized bioinformatics-connected approaches including cloning, Sanger sequencing, molecular analysis, DNA microarray, high-throughput, and metagenomic sequencing technologies. 

Despite the great success over the last decades in the detection limit of eco-genetic tools of EVs, techniques that are straightforward, simple, low-cost, and broadly applicable are still absent. In addition, the co-existence of co-culture of different viral pathogens is also a hurdle that hinders rapid virus detection and/or diagnosis. Shortcomings are present in most techniques, and they need to be amended, including those in cell culture-based, immunological, molecular-based, and other up-to-date unconventional methods. 

Although they are high-cost techniques compared with conventional methods and biosensors, microarray, and metagenomics-based techniques can successfully recognize and precisely detect many new EVs in recent decades and seems to be an auspicious approach. To this point, we are convinced that the implementation of these advanced techniques will in time enrich our understanding about the epidemiology of EVs in aquatic ecosystems and will provide indirect assistance to vaccine development recommendations against EVS and their emerging diseases. Nevertheless, the high cost of these technologies remains an obstacle for low-resourced areas and developing countries experiencing substantial pollution with an unprecedented health and economic crisis. This necessitates flexible and intensive international collaboration within the “one world one health” approach among developed and developing countries to establish the minimum essential virus detection techniques in the central laboratories of the developing countries, enabling them to have their own alarming system for emerging and reemerging water-related viral pathogens of either epidemic or pandemic potential. Moreover, the combination of two or more conventional detection methods is recommended at the moment for developing countries to ensure better viral pathogen detection with affordable cost.

## Figures and Tables

**Table 2:** Enteric viruses (EVs) and recommended cell culture systems for propagation.

Virus	Cell Line	Origin	Ref.
Avian influenza viruses	SPF-ECE	Specific pathogen-free embryonated chicken egg	[61]
	MDCK	Madin–Darby Canine Kidney cell	
	Vero	African green monkey kidney cell	
Adenovirus	A549	Human lung carcinoma cell	[62]
	PK-15	Porcine kidney epithelial cell	
Astrovirus	HEK	Human embryo kidney	[63]
	Caco-2	Human colorectal adenocarcinoma cell	
	A549	Human lung carcinoma cell	
Bocavirus	Caco-2	Human colorectal adenocarcinoma cell	[64]
	HEK293	Human embryonic kidney cell	[65]
	HTEpC	Human trachea epithelial primary cell	[66]
Coxsackievirus	HeLa	Human cervical cancer cell	[67]
Coronavirus	MRC-5	Human fetal lung fibroblast cell	[68]
	Vero-E6	African green monkey kidney cell	[69]
Enterovirus	RD	Human muscle tissue	[70]
HEV	A549	Human lung carcinoma cell	[71]
HAV	Caco-2	Human colorectal adenocarcinoma cell	Reviewed in [72]
	HepG2-N	Human hepatoma	
	Huh-7	Hepatocarcinoma cell	
	MRC-5	Human fetal lung fibroblast cell	
	Vero	African green monkey kidney cell	
Norovirus	BJAB	Human B cell lines	[73]
	iPSC–derived IECs	Human induced pluripotent stem cell	[74]
Rotavirus	MA-104	African green monkey epithelial cell	[75]
	HT-29	Human colon carcinoma cell line	[76]
	Caco-2	Human colorectal adenocarcinoma cell	[77]
Reovirus	Vero	African green monkey	[78]
Sapovirus	LLC-PK1	Porcine kidney cell	[67]

**Table 3:** Isothermal nucleic acid amplification-based assays.

Technique	Principle	EVs to Detect	Ref.
Nucleic acid sequence-based amplification (NASBA)	- Recommended for RNA detection - Demands the activity of the reverse transcriptase (RT), T7 RNA polymerase and then RNase H, consequently - Two primers are required: 1st binds to T7 RNA polymerase and 2nd binds to the cDNA formed	Human adenovirus and echovirus	[58,99]
Loop-mediated isothermal amplification (LAMP)	- Includes an isothermal amplification of a targeted sequence in a loop mediated displacement - Utilizes a set of 4–6 special primers - Initial denaturation of the template is not necessary	Noroviruses and swine acute diarrhea syndrome-coronavirus	[58,100,101]
Single primer iso-thermal amplification (SPIA)	- A single, target-specific primer containing 3′-DNA sequence portion and 5′-RNA sequence portion is required - The amplification system includes a DNA polymerase with efficient strand-displacement activity, RNase H, and blocker. - Can be visualized using polyacrylamide gel electrophoresis, or by incorporating SYBR Green II	Human norovirus	[58,102]
Recombinase polymerase amplification (RPA)	- Isothermal amplification using recombinase polymerase amplification (RPA) to the reverse transcriptase enzyme, enabling the detection of RNA and DNA - A separate cDNA step is not required. - RPA can be performed using simpler equipment than PCR	Human norovirus, avian influenza virus and bovine viral diarrhea virus (BVDV)	[58,103,104,105]

**Table 4:** Merits and demerits of viral pollution detection method.

Method	Advantages	Disadvantages	Ref.
**Electron microscopy**	- Allows fast morphological identification - Does not require special considerations and/or reagents	- Unsuitable as a screening method - Does not provide information about virus infectivity or genotype	[121]
**Cell culture**	- Provides information about viral infectivity	- Time-consuming - Cell lines are not available for all viruses - Not effective in case of slow-growing and non-replicating viruses - Expensive than other methods	[16]
**Immunological methods (e.g., ELISA and latex agglutination technique)**	- Simplify the detection method - Shorten detection time - Less labor intensive than other conventional methods	- False positives are often occurred - Unable to determine viral infectivity - The successful implementation of immunological detection techniques in the water field often integrated with conventional cell culturing methods or microscopy	[122]
**Polymerase chain reaction (PCR)**	- Decrease the time for detection (4–6 h) - Sensitive and specific - Can be multiplexed	- Inability to differentiate between infectious/active and non-infectious viral particles in the sample - Can be affected by inhibitor substances - Non-quantitative method, “qualitative”	[16]
**Nested and semi-nested PCR**	- Decrease the time for detection - Sensitive compared with PCR	- Contamination risk in second round of PCR	[89]
**Multiplex PCR**	- Able to detect several viruses in one reaction - Decrease the time for detection and overall detection cost	- Decrease the sensitivity - May produce non-specific product	[123]
**Real-time PCR (rt-PCR or qPCR)**	- Quantitative - Sensitive - Specific - Can be multiplexed - Results can be obtained within 2–4 h	- Does not differentiate between infectious and noninfectious viruses - Prone to PCR inhibition - Expensive reagents and equipment	[16]
**ICC-PCR**	- Highly efficient to detection of infectious viruses than cell culture, decrease the time for detection	- Slow compared with PCR methods - Labor intensive	[124]
**Digital PCR**	- Does not require standard curve - More sensitive than qPCR - Less affected by PCR inhibitors in water specimens	- Expensive platform - Limited dynamic range of detection - Lower sample capacity compared with qPCR format	[16,94]
**Isothermal nucleic acid amplification-based assays**	- Rapid compared with PCR - Can be multiplexed - Does not require a thermal cycler - Portable and easy-to-use detection method - Suitable for a laboratory with basic instruments	- Require special knowledge of primer design - Require special polymerase or recombinase enzymes - Sample pretreatment is required - RNA handling may require attention	[16]
**Biosensors**	- Versatile and fast - Can recognize and bind different pathogens with high affinity and specificity - Greater long-term storage stability - Potential reusability - Easy-to-use - Requires a very low amount of the sample - Cheaper and easy disposability	- Mostly qualitative, “no quantification”	[125]
**Microarrays**	- Can detect multiple viral targets - Powerful diagnostic tool	- Requires significant bioinformatics knowledge - Random amplification is a prerequisite - Sensitivity is uncertain for environmental waters - Qualitative method, “non-quantitative” - Does not determine the infectivity of the viral particles in the sample	[16]
**Metagenomics or next-generation sequencing (NGS)**	- Does not require sample culturing or viral genome cloning - Can detect novel or unknown emerging viruses	- Removal of cellular organisms is a prerequisite - PCR preamplification is required - Multifaceted process and demands special expertise in sequence data analysis - Absolute quantification is not provided - Machine is costly - Does not determine the infectivity of the viral particles in the sample	[16]
**ChIP-Seq analysis**	- Does not require sample culturing or viral genome cloning	- Limited to studying proteins that are bound to genetic materials rather than diagnosis - Does not determine the infectivity of the viral particles in the sample - Expensive and time-consuming	[126]

## Data Availability

Not applicable.
